# Hysteretic Behavior in Voltage-Gated Channels

**DOI:** 10.3389/fphar.2020.579596

**Published:** 2020-11-02

**Authors:** Carlos A. Villalba-Galea, Alvin T. Chiem

**Affiliations:** Department of Physiology and Pharmacology, Thomas J. Long School of Pharmacy, University of the Pacific, Stockton, CA, United States

**Keywords:** hysteresis, voltage-gated channels, voltage-sensing domain, voltage-sensitive phosphatases, modal gating, mode shift, voltage-sensing domain relaxation

## Abstract

An ever-growing body of evidence has shown that voltage-gated ion channels are likely molecular systems that display hysteresis in their activity. This phenomenon manifests in the form of dynamic changes in both their voltage dependence of activity and their deactivation kinetics. The goal of this review is to provide a clear definition of hysteresis in terms of the behavior of voltage-gated channels. This review will discuss the basic behavior of voltage-gated channel activity and how they make these proteins into systems displaying hysteresis. It will also provide a perspective on putative mechanisms underlying hysteresis and explain its potential physiological relevance. It is uncertain whether all channels display hysteresis in their behavior. However, the suggested notion that ion channels are hysteretic systems directly collides with the well-accepted notion that ion channel activity is stochastic. This is because hysteretic systems are regarded to have “memory” of previous events while stochastic processes are regarded as “memoryless.” This review will address this apparent contradiction, providing arguments for the existence of processes that can be simultaneously hysteretic and stochastic.

## Introduction

When thinking about a physical or chemical system that responds to a “stimulus,” it is commonly assumed that such system would display a constant activity-vs.-stimulus relationship. For instance, imagine a ligand-activated receptor with one binding site and an affinity of 1 µM for an agonist ligand. Adding 100 µM of such ligand will result in 99% of ligand-bound receptors, leading to their activation. On the other hand, decreasing the concentration to 0.01 µM of the agonist will result in less than 1% of ligand-bound receptors, deactivating these proteins. In theory, the level of activity of the receptor will only depend on the current concentration of the agonist ligand. This will always be the case regardless of how the ligand concentration changes. In other words, the receptor does not “remember” what happened before the ligand was at the current concentration, what the concentration of the ligand had been in the past, or what levels of activation were previously reached. This lack of “memory” of the receptors is the consequence of the ligand binding and activation being stochastic processes, unaffected by previous exposures of the receptor to the ligand.

In the case of voltage-gated channels, an analogous scenario is established when considering that the electrical field across the plasma membrane acts as the “stimulus” that drives channel activation and deactivation. As an example, let us consider a tetrameric voltage-gated cation-selective channel that has three effective sensing charges per subunit and that reaches half of its maximum activity at 0 mV. Assuming that it does not inactivate, this channel will be at 0.1% of its maximum activity when the membrane potential is −60 mV, while reaching about 99% of its maximum at a membrane potential of +40 mV. These activity levels will be reached at the respective membrane potential levels regardless of the history of the channel’s activity. This voltage sensitivity remains unaltered over time.

Like in the example of the hypothetical receptor discussed above, it is commonly thought that the activity-vs.-voltage relationship that describes the behavior of voltage-gated channels is a static function of the membrane potential. However, a growing body of evidence shows that the electrical sensitivity of voltage-gated channels can be dynamic rather than static. This dynamic character of the voltage dependence seems to be rooted in the hysteretic behavior of channels and has important consequences on the physiology and pharmacology of these proteins. Therefore, hysteresis seems to play a critical role in the generation and modulation of electrical signal events in neurons, muscles, and other excitable tissues.

## How Could Hysteresis Affect Electrical Signaling in Cells?

Let us consider the simple case of an excitable cell that expresses a prototypic sodium-selective voltage-gated (Na_V_) channel and a prototypic potassium-selective voltage-gated (K_V_) channel. In addition, let us also consider that the cell has a basal, non-voltage-dependent conductance that is mostly selective for K^+^ ions. Upon reaching a threshold potential following a depolarizing stimulus, the action of Na_V_ channels will further depolarize the membrane. Then, while Na_V_ channels inactivate, the activity of K_V_ channels will start to repolarize the membrane, counteracting the Na_V_-driven depolarization. As the membrane gradually returns to more negative voltages, K_V_ channels start to close. This decreases the voltage-dependent K^+^ conductance of the membrane as it crawls back to its resting potential. In this case, the basal conductance of the membrane becomes critical in completing the repolarization process. This is the classical view of the development of an action potential (AP), derived from the outstanding work of Hodgkin and Huxley (1952).

On the other hand, in a more modern view, the voltage dependence of K_V_ channels is dynamic and shifts to more negative potentials following activation. In this case, hysteresis manifests as Dynamic Voltage Dependence (DVD) of K_V_ channel activity and makes the repolarization a more robust process. This is because transiently shifting the K_V_ channel’s voltage dependence to more negative potentials would provide a steadier K^+^ conductance as the membrane potential nears its resting values and beyond. Therefore, it is likely that this hysteretic behavior is essential for the generation and stability of cellular electrical signaling. Hysteresis in their behavior can make the deactivation of K_V_-related conductance more resilient to closing at resting and to developing hyperpolarized potentials during repolarization.

Based on the previous example, it is clear that hysteresis is an important property of voltage-gated channels. Thus, understanding the mechanism of this phenomenon will be essential for a comprehensive study of channel activity. To start, this review will provide a definition of hysteresis in terms of voltage-gated channel activity.

## What Is Hysteresis?

The term hysteresis derives from the Greek ὑστέρησις, meaning “lagging behind.” The term was used in 1881 by Sir James Alfred Ewing to describe the effect of magnetization on current induction in a conductor (Ewing, 1882). Subsequent studies showed one of today’s best-known examples of hysteresis: the magnetization of a ferromagnetic material. Let us consider a sample of a ferromagnetic material (e.g., iron, a screwdriver) and impose a magnetic field across the sample. This results in magnetization of the sample, which will remain magnetically polarized even when the external field is removed. This means that the material “remembers” that it was magnetized. If the sample is allowed to rest for a long time or if it is heated up, then the magnetization will be lost. Overall, once the external field is removed, the material will still remember that it has been magnetized and remain magnetized for a finite time.

Extending the previous example, let us consider the following steps in a thought experiment: 1) apply a magnetic external field, 2) remove the field, and 3) reapply the external magnetic field in the reverse direction. Respectively, the outcomes of each step in this experiment will be that: 1) the material will be magnetically polarized, 2) it will remain magnetized, and 3) the magnetization will be reverted. As it can be seen by the outcome of step 2, the material will resist giving up its magnetization, even when the external magnetic field has been removed. Yet, this magnetic polarity can be reverted by applying a magnetic field in the reverse direction. Further extending this example, let us consider cyclically imposing the external magnetic field in one direction and then the reverse direction. Because the material resists giving up its polarity, what would be observed is that the amplitude and direction of the magnetization will be trailing behind with respect to the external magnetic field. The observance of this lagging response resulted in the coinage of the term “hysteresis.”

## Physiological Role of Hysteresis

Ion channels are a critical component of the plasma membrane in all known living beings. These proteins are responsible for the generation of electrical activity in cells, which is essential for many biological processes. The physiological relevance of hysteresis remains elusive. However, the voltage dependence of several K_V_ channels shifts to more negative potentials following activation, displaying a behavior analogous to that of ferromagnetic materials. This lagging change in voltage dependence can be seen as an “on-demand” fine-tuning of channels’ responsiveness during the generation of electrical signals. So, K_V_ channel closing happens when the membrane potential goes to further negative voltages, guaranteeing that the membrane’s K^+^ conductance will remain activated during repolarization.

Another interesting consequence of DVD is that the voltage dependence for activation is shifted towards more positive potentials while the K_V_ channels are closed at the resting potential. This may likely facilitate the triggering of an electrical signal by keeping the voltage-dependent K^+^ conductance of the membrane relatively low as the depolarizing conductances are initiated (i.e., Na^+^ and Ca^2^^+^ conductance). Therefore, DVD may constitute a molecular strategy that has evolved to facilitate the initial triggering of fast electrical signals and to ensure their prompt termination. Furthermore, if Na_V_ channels were to display hysteresis, this will also contribute to preventing reactivation of the Na^+^ conductance during the inactivation process (since recovering from inactivation of the Na_V_ channels would require further repolarization of the membrane).

The observed “inertia” in the responsiveness of K_V_ channels, which is driven by changes in their electrical sensitivity, resembles that of ferromagnetic materials displaying hysteresis. In this case, the term “sensitivity” refers to the range of potential at which the channel changes, rather than the amount of change. So, it can be argued that hysteresis in the activity of K_V_ channels can make them harder to open when they are closed and harder to close when they are open. One example of this behavior is that of the potassium channel K_V_3. These channels have a half-maximum potential for current activation above 0 mV (Kaczmarek and Zhang, 2017). Also, K_V_3 channels have very fast deactivation kinetics that make them particularly suitable for high frequency firing of APs (Rudy et al., 1999; Rudy and McBain, 2001; Lien and Jonas, 2003; Kaczmarek and Zhang, 2017). In addition to these properties, it has been shown that K_V_3.1b channels display a remarkable shift in their voltage dependence for sensing charge movement (gating currents) of about −60 mV following activation (Labro et al., 2015). This shift favors channel opening during repolarization. This shift in the voltage-dependence of the channels results in a notable decrease in the deactivation rate kinetic of their conduction, guaranteeing a quick and efficient repolarization of the membrane (Labro et al., 2015).

In summary, hysteresis in the activity of voltage-gated channels can have remarkable consequences in the generation of APs. Thus, comprehensive study on the fundamentals of this process could lead to a novel perspective on the understanding of voltage-gated channel activity in cellular electrical signaling.

## Candidate Mechanisms for Hysteresis in S4-Voltage-Sensing Domain Proteins

The activity of ion channels can be defined by at least the following two general parameters: 1) the probability of channels to be open and 2) their ability to conduct and discriminate ions. This review will focus on the first of these general parameters.

Voltage-gated channels are so called because the probability of these channels to be open (open probability, P_O_) is a function of the difference in electrical potential across the membrane. There are several types of voltage-gated channels. Here, the focus of the review will be on channels containing a voltage-sensing domain (VSD) made of four transmembrane helical segments (S1–S4), with the fourth segment (S4) bearing the main voltage-sensing charges. This type of voltage-gated channels are referred to as “S4-based voltage-gated channels,” or “S4-VSD channels.”

Evidence for hysteresis in S4-VSD channels dates from the early 1980s, when it was described for the sodium conductance that is observed in the squid giant axon (Bezanilla et al., 1982). It was shown that the voltage dependence for sensing charge movement is dependent on the holding potential, shifting to more negative potentials when the membrane was held at 0 mV instead of −70 mV. Analogous to the case of magnetization of a ferromagnetic material, the return of gating charges back to their resting state required bringing the membrane to more negative potentials when the membrane was initially held at 0 mV.

Similar observations were made over a decade later with the potassium-selective voltage-gated channel known as “Shaker” (Olcese et al., 1997; Lacroix et al., 2011). Like that of the squid axon’s sodium conductance, the voltage-dependence for charge movement of Shaker was sensitive to the holding potential. In this case, the voltage dependence for gating charge movement was displaced approximately 25 mV towards more negative voltages when the holding potential was set at 0 mV instead of −90 mV (Olcese et al., 1997). Later research showed that the change in voltage dependence was overestimated due to a remarkable decrease in the rate of deactivation (Lacroix et al., 2011). Nonetheless, at that time, it was proposed that the channels could either be in a “permissive” or “reluctant” conformation (Olcese et al., 1997). In the “permissive” conformation, the channel was able to be activated and charges were readily moved. In the “reluctant” conformation, the channel was inactivated and charge movement was slow, requiring further hyperpolarization of the membrane. Adopting the “reluctant” state was associated with the slow inactivation of Shaker (Olcese et al., 1997). As the inactivated state is reached after the open state, it becomes more thermodynamically stable. This would make the open state a “transitory” or “meta-stable” state. From this idea, it can be concluded that bringing the channel out of a stable state could require more energy than was needed to move the charges in the first place (Villalba-Galea, 2017).

**FIGURE 1:**
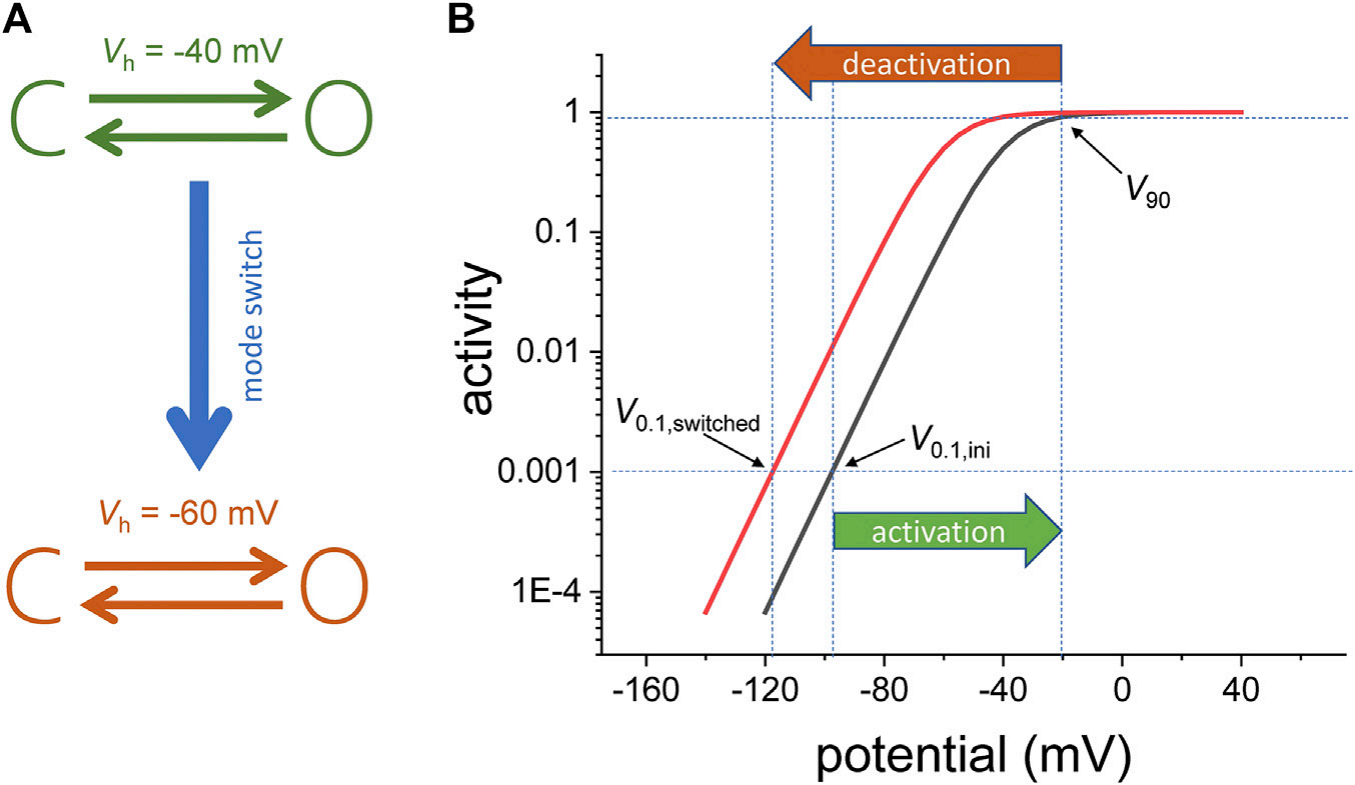
**(A)** Voltage dependence model for a channel consisting of one closed (C) and one open (O) state. Following activation, the two-state model switches from one mode of activity to another. For the initial mode, the voltage the half-maximum potential (*V*_h_) is −40 mV. For the final mode, V_h_ is −60 mV. **(B)** Semi-log plot of the activity (fraction of the open channels). Due to the mode switch, the change in the potential needed to drive the fraction of open channels from 0.001 to 0.9 (|*V*_90_−*V*_0.1,ini_|) is smaller in magnitude that the change in potential required to bring the fraction of open channels back to 0.001(|*V*_0.1,switched_−*V*_90_). The indices 0.1 and 90 refer to 0.1 and 90% of the channel population, respectively.

Another interesting aspect of Shaker is that the channels in the “permissive” and “reluctant” conformations are considered to be in separate interconvertible populations, where the fraction of channels in each of those populations are dependent on the holding potential (Olcese et al., 1997). The fact that there are two populations means that the activity of the VSD can adopt to “modes of operation.” To illustrate this idea, let us consider a voltage-dependent channel that has two states: closed and open (Figure 1A, top). Following activation from an initial holding potential (V_0.1,ini_) to a given potential that will bring it to 90% of activity (V_90_), the channels can switch to another “mode of activity” with a different sensitivity to the membrane potential (Figure 1A, bottom). In this new mode, the membrane must be set at a potential (V_0.1,switched_) that is more negative than V_0.1,ini_ in order to bring the channel to the initial activity level (Figure 1B).

For many years, changes in the voltage dependence for charge movement in Shaker was linked to slow inactivation, which was thought to be due to conformational rearrangements in the pore domain. However, studies of charge movement in the voltage-sensitive phosphatase isolated from *Ciona intestinalis* (Ci-VSP) provided evidence demonstrating that DVD can be a phenomenon intrinsic of a S4-VSD (Villalba-Galea et al., 2008; Akemann et al., 2009; Villalba-Galea et al., 2009b). The voltage-controlled enzyme Ci-VSP is a dimeric protein that is made of a S4-VSD that is homologous to that of voltage-gated channels (Murata et al., 2005; Murata and Okamura, 2007; Sakata and Okamura, 2019). The VSD is linked to the phosphoinositide phosphate domain through a phospholipid/phosphoinositide binding motif (Villalba-Galea et al., 2009a; Kohout et al., 2010; Hobiger et al., 2012; Hobiger et al., 2013). The action of the VSD confers voltage sensitivity to the VSP’s enzymatic activity through a mechanism that remains under debate (Villalba-Galea, 2012a; Villalba-Galea, 2012b; Sakata and Okamura, 2019).

Under voltage-clamp, sensing currents can be observed during the activation of a VSD. These currents are due to the movement of sensing charges in the VSD, analogous to gating currents in voltage-gated channels (Sakata and Okamura, 2019). In the case of Ci-VSP, the voltage dependence for charge movement shifts about −50 mV when holding the membrane potential at +80 mV instead of −60 mV or more negative voltages. Furthermore, deletion of the phosphoinositide phosphate domain of Ci-VSP causes a larger shift in voltage dependence for sensing charge movement following relaxation. Such shift that nearly doubled that of the intact enzyme (Villalba-Galea et al., 2008). This observation indicates that an isolated VSD can shift its voltage dependence without being coupled to another domain. Determining whether the interaction between the two VSDs causes a shift in voltage dependence has yet to be ruled out. Yet, more recent studies expressing the isolated VSD of Shaker shows that this type of domain can display an intrinsic hysteretic behavior (Zhao and Blunck, 2016).

## Voltage-Sensing Domain Relaxation

Another important aspect of the DVD concept is that changes in the voltage-dependence do not occur instantaneously. Instead, the displacement of the activity-vs.-potential relationship towards negative voltages is a process that can take time (Villalba-Galea et al., 2008; Villalba-Galea et al., 2009b; Villalba-Galea, 2017). In the case of Ci-VSP, the shift in voltage dependence has been proposed to occur through a process termed “VSD relaxation” (Villalba-Galea et al., 2008). In Ci-VSP, VSD relaxation can take seconds, indicating that it continues even after sensing currents have ended. This suggests that VSD relaxation is an intrinsically voltage-independent process.

VSD relaxation is not unique to Ci-VSP or Shaker—it can be observed in other S4-VSD proteins such as Hyperpolarization-activated Cyclic Nucleotide-gated (HCN) channels (Mannikko et al., 2005; Elinder et al., 2006; Bruening-Wright and Larsson, 2007) and the human Ether-à-go-go-Related Gene channel (Pennefather et al., 1998; Piper et al., 2003; Thouta et al., 2017; Shi et al., 2019; Shi et al., 2020). In these cases, VSD relaxation has been proposed to play an important role in the remarkable hysteretic behavior observed in these proteins. For HCN channels, hysteresis is manifested as a process called “mode shift” (Mannikko et al., 2005; Elinder et al., 2006). As the term implies, the concept is that channels can adopt one of at least two discrete modes of activity as a function of the membrane potential. The same idea seems to apply to VSD relaxation. For Shaker, the charge-vs.-potential (Q-V) curves show two populations of charges moving with distinct voltage sensitivity (Olcese et al., 1997). Furthermore, the fraction of the total charge in each mode is a function of the holding membrane potential. This indicates that modes of activity in a VSD also are likely discrete sets of states, that channel activity can have more than one of these modes of activity, and that channels can adopt any of these modes one at a time.

## Voltage-Sensing in S4-Voltage-Sensing Domain Channels

The prototypic S4-based voltage sensor consists of four transmembrane (S1–S4) segments with S2, S3, and S4 segments containing charged residues (Islas, 2016). Typically, S2 and S3 segments bear 1 or 2 negative charges each, in the form of aspartate or glutamate residues. In contrast, the S4 segment contains positive charges in the form of arginine, lysine, and histidine residues. The current understanding is that the VSD charges, mainly those of the S4 segment, are displaced as a function of the membrane potential. In other words, the electrical gradient across the membrane does work on the VSD charges, leading to the rearrangement of the VSD. On this idea, let us consider the electrical field across the plasma membrane that does electrical work on the sensing charges, which can be calculated as:

$$W_{ELECT}=\underset{i}{\sum }\overset{b_{i}}{\underset{a_{i}}{\int }}Q_{i}E\left(r_{i}\right)dr$$ (1)

This means that the total electrical work (*W*_*ELECT*_) is the sum of the work done on each charge. The work done by the electrical field on the *i*-th charge (*Q*_*i*_) is given by the integral of the magnitude of that charge multiplied by the electrical field *E*(*r*_*i*_) as a function of the position of the charge (*r*_*i*_). The integral is calculated between position *a*_*i*_ and *b*_*i*_, which respectively represent the initial and final positions of the i-th charge.

$$W_{ELECT}=\overset{b_{1}}{\underset{a_{1}}{\int }}Q_{1}E\left(r_{1}\right)dr+\overset{b_{2}}{\underset{a_{2}}{\int }}Q_{2}E\left(r_{2}\right)dr+\ldots +\overset{b_{n}}{\underset{a_{n}}{\int }}Q_{n}E\left(r_{n}\right)dr$$ (2)

This equation seems difficult to resolve, as the electrical field E is a function of the position of the charge. However, it can be simplified when making some assumptions on the structure and functioning of a prototypic S4-VSD. These assumptions are as follows:

In terms of the VSD structure, the prototypical S4-based VSD consists of the four transmembrane helices arranged in a bundle that forms two crevices—one on each side of the membrane. These two crevices are separated by a small hydrophobic volume (Starace et al., 1997; Starace and Bezanilla, 2001; Starace and Bezanilla, 2004; Ahern and Horn, 2005). This region is commonly referred to as the “hydrophobic plug,” also known more precisely as the “hydrophobic gasket.” Structural evidence shows that the hydrophobic gasket is a few Angstroms thick, only hosting one charge residue at a time. Consequently, it can be assumed that some of the S4 segment charge residues can cross the entire electrical field.

In terms of the VSD functioning, several S4-VSD proteins have been shown to have a conductance through their VSD when some of their residues are replaced. To illustrate this, let us consider the case of Shaker. Replacing the S4-segment arginine located at positions 362, 365, 368, and 371 with histidine results in voltage-dependent proton conductance through the channel’s VSD itself (Starace et al., 1997; Starace and Bezanilla, 2001; Starace and Bezanilla, 2004). In the particular case of mutation R362H and R371H, proton currents are observed only at the resting and activated conformations of the VSD, respectively. In contrast, the mutants R365H and R368H mediate a proton conductance when the VSD is residing in intermediate states between the fully rested or fully activated positions. These implies that residues R362 through R371—hereafter R1 through R4—can reside in the hydrophobic gasket, having access to both the intracellular and extracellular side of the membrane. Furthermore, this also implies that R365H and R368H—hereafter R2 and R3—cross the entire field. Therefore, in a channel like Shaker, the total number of charges per VSD on which the electrical field is acted on is between 2 and 4 elementary charges. Using Eq. 2, we can state that,

$$W_{ELECT}=\overset{b_{1}}{\underset{a_{1}}{\int }}Q_{1}E\left(r_{1}\right)dr+\overset{b_{2}}{\underset{a_{2}}{\int }}Q_{2}E\left(r_{2}\right)dr+\overset{b_{3}}{\underset{a_{3}}{\int }}Q_{3}E\left(r_{3}\right)dr+\overset{b_{4}}{\underset{a_{4}}{\int }}Q_{4}E\left(r_{4}\right)dr$$ (3)

Since each charge is an elementary charge, all Q_i_ are equal and will be called Q_e_. Replacing and rearranging Eq. 3 yields:

$$W_{ELECT}=Q_{e}\left(\overset{b_{1}}{\underset{a_{1}}{\int }}E\left(r_{1}\right)dr+\overset{b_{2}}{\underset{a_{2}}{\int }}E\left(r_{2}\right)dr+\overset{b_{3}}{\underset{a_{3}}{\int }}E\left(r_{3}\right)dr+\overset{b_{4}}{\underset{a_{4}}{\int }}E\left(r_{4}\right)dr\right)$$ (4)

If the field across the hydrophobic gasket is assumed to be constant, each of the terms within the parenthesis would be:

$$\overset{b_{i}}{\underset{a_{i}}{\int }}E\left(r_{i}\right)dr=\;\delta_{i}\Delta V_{M}$$ (5)

In Eq. 5, $\Delta V_{M}$ is the difference between the initial and the final membrane potential; δ corresponds to the fraction of the electric field that each charge crossed. In the case of charges R2 and R3, assume that they both cross the entire electric field, resulting in the term $\delta_{i}$ equal to 1. In the case of charges R1 and R4, the term would be within 0 and 1. Taking these assumptions into account, replacing Eq. 5 into Eq. 4 yields:

$$2Q_{e}\Delta V_{M}<W_{ELECT}<4Q_{e}\Delta V_{M}$$ (6)

To put this in the context of a typical voltage-clamp experiment, let us consider that the membrane is held at −90 mV and an activating pulse is applied to +10 mV. This will make $\Delta V_{M}$ equal to +100 mV. Note that the range of potential considered for this example allows for a meaningful displacement of the sensing charges that are driven by the electric field—if the charges do not move, then no electrical work is done. The activation of four VSDs will result in an overall electrical work ranging between 7.71 × 104 and 1.15 × 105 J/mol (between 18 and 37 kCal/mol). Since both R1 and R4 can gain access to both the intracellular and extracellular crevice of the VSD, it is unproven but likely that these residues cross at least half of the electric field. For this reason, it can be stated that the electric field does work on the equivalent of three elementary charge per VSD, meaning that the total electrical work is approximately $3Q_{e}\Delta V_{M}$. Consistently, experimental data, kinetic modeling, and estimation of the free energy show that the activation of Shaker involves at least 13 charges, or 3.25 charges per VSD. This translates into a total work of about 30 kCal/mol during activation (or deactivation) of the four VSDs.

To put this in perspective, let us consider that gating currents for Shaker are a simple two-state system, with R being the resting state and A being the activated state. Assuming this system, we calculate the equilibrium constant (also known as the Boltzmann distribution) for R and A as follows:

$$k_{eq}=\frac{A}{R}=e^{\frac{-W_{ELECT}+\Delta G_{C}}{k_{B}T}}$$ (7)

Assuming that the free energy of activation Δ*G*_*C*_ is −14 kCal/mol and that there are 3.25 charges per VSD, the ratio R/A that is given by the Boltzmann distribution (Eq. 7) will be 8.6 × 10−^4^ and 4.2 × 102 at −100 and 0 mV, respectively. This involves a 5.7-order-of-magnitude increase in the activity of each VSD. Putting this observation in more familiar terms, the fraction of activated VSD can be calculated as,

$$f_{A}=\frac{A}{A+B}\;\;\;\Rightarrow \;\;\;f_{A}=\frac{k_{eq}}{k_{eq}+1}$$ (8)

Replacing and rearranging Eq. 7 into Eq. 8 yields a Fermi-Dirac distribution:

$$\frac{k_{eq}}{k_{eq}+1}=\frac{1}{1+e^{\frac{-W_{ELECT}+\Delta G_{C}}{k_{B}T}}}$$ (9)

Because *W*_*ELECT*_ is proportional to the sensing charge (Eq. 6) and $\Delta G_{C}$ is proportional to the total charge, Eq. 9 can be re-written as,

$$f_{A}=\frac{1}{1+e^{\frac{-zQ_{e}\left(\Delta V-\Delta V_{C}\right)}{k_{B}T}}}$$ (10)

Notice that the Fermi-Dirac distribution is transformed into what is commonly referred to as the “Boltzmann distribution.” It is important to highlight that the term “Boltzmann distribution,” which has been used in electrophysiology for many years, is a misnomer. The ratio between the active and resting states is the correct way to refer to a Boltzmann distribution for a 2-state model (like in Eq. 7). The fraction of the distribution representing the open state is the correct way to refer to a Fermi-Dirac distribution (like in Eq. 10).

The exponential term $zQ_{e}\Delta V_{C}$ in Eq. 10 represents the magnitude of the free energy of activation for a VSD, where z represents the number of elementary charges and Qe represents the elementary charge. This is only true if the VSD behaves as a two-state system—an elegant procedure that has been described by Chowdhury and Chanda (2012). The basic Boltzmann and Fermi-Dirac distributions alluded here formally describe systems with two discrete states. However, in the case of voltage-gated channels, the existence of multiple interconnected states has been well-established. Thus, describing their behavior requires more elaborate functions. Yet, these equations can provide meaningful approximations when using a suitable charge value. So, the change in free energy following VSD relaxation is given by:

$$\Delta G_{RELAX}=zQ_{e}\left(\Delta V_{C,ACT}-\Delta V_{C,RELAX}\right)$$ (11)

According to Eq. 11, when considering a total of 3.25 elementary charges per VSD, there is a change of −2.98 kCal/mol for every 10 mV of voltage-dependence displacement.

## Dynamic Kinetics and Hysteresis

The HCN channel isolated from the sea urchin *Strongylocentrotus purpuratus* (spHCN) shows activity with at least two modes, which are populated as a function of the holding potential and a function of the duration of activation (Mannikko et al., 2005; Bruening-Wright and Larsson, 2007). In either mode, channels undergo a mode shift. Another important observation is that the deactivation becomes slower as the channels are kept activated. Likewise, the activation becomes faster as a function of how recently they were last activated. In both cases, “remembering” the event in the near past conditions the behavior of the channel—a hallmark of hysteresis. This shows that it is possible to observe a voltage-dependent conformational change in the channel, which can bring it to a short-lived transient state referred to as a “meta-stable state.” Particularly, in channels with slow inactivation, the open/activated set of states reached following activation corresponds to meta-stable states. On the other hand, prolonging the activation of the channels lead to a set of inactivated or low-activity states which corresponds to stable states.

From a meta-stable state, a channel can transition into a more stable state, reaching steady state. To illustrate this idea, consider a deactivated channel in steady state at a given holding potential (e.g., human Ether-à-go-go-Related Gene and −90 mV or spHCN at 0 mV). Then, change the potential to a voltage that activates the channel. Focusing on the gating currents, the VSD will first be brought out of its steady state conformation arrangement as the electric field does work on the sensing charges. If this process is thermodynamically reversible in infinitesimal steps, then returning to its original resting state will occur with no changes to its surroundings. Thus, the path back to the resting state will be unaltered. This is likely not the case for voltage-gated channels, because the sudden change in the electrical field (e.g., ∼1 × 108 V/m for a 100-mV change) would result in a rapid rearrangement of the protein—a product that would not be in thermodynamic equilibrium (approx. 1 megawatts, assuming a 20 ms charge movement). In addition, it is likely that many intramolecular and intermolecular interactions are to take place, given that the channel and its surroundings are composed of condensed matter. In this view, the activation could lead the channel into a meta-stable open conformation, from which it would eventually “relax” into a stable arrangement. If this is the case, it would then be expected that deactivation will also be slower from the stable state compared to the meta-stable state.

Although the existence of such conformations remains a matter of debate, electrophysiological studies have provided examples of supporting evidence. In fact, several voltage-gated channels demonstrate this feature in their respective activity. For instance, prolonging the activation of HCN, Shaker, and K_V_3.1b leads to a decrease in the rate of deactivation for both gating currents and ionic currents (Mannikko et al., 2005; Elinder et al., 2006; Bruening-Wright and Larsson, 2007; Lacroix et al., 2011; Labro et al., 2012; Priest et al., 2013; Labro et al., 2015; Zhao and Blunck, 2016). Others, like K_V_7.2 and K_V_7.2/K_V_7.3 channels, undergo remarkable decreases in their rate of deactivation as a function of activation (Corbin-Leftwich et al., 2016). This indicates that the activated/open state that is observed in the study of any voltage-gated channel immediately after depolarization may not be in a thermodynamically stable state.

## Implications of Hysteresis to the Current Understanding of S4-Voltage-Sensing Domain Protein Function

One remarkable instance is K_V_3.1b, which undergoes a fast VSD relaxation that enables a resurgent current during deactivation (“hooked tail”) (Labro et al., 2015). Under voltage-clamp, activation by a pulse to positive values readily activates the channel. Then, returning to a moderate negative potential (−40 or −50 mV) results in a transient increase in the conductance. This occurs because the VSD quickly relaxes as it is activated, shifting its voltage-dependence to more negative potentials. This phenomenon has strong physiological implications because the rising of a “hooked tail” current during deactivation yields a transient increase in repolarizing power, securing the repolarization of the plasma membrane even after a brief depolarization (Labro et al., 2015). From a generalizing angle, it can be said that this kind of mechanism prevents K_+_-selective voltage-gated channels from rapidly closing, since their own activity repolarized the membrane and caused them to deactivate themselves.

As another example, mode shift in HCN channels seems to be essential to their role in pace-making. In essence, the activation of HCN currents leads to the depolarization of the membrane and eventual triggering of an electrical event (e.g., an AP in the sinoatrial node) (Elinder et al., 2006). As the AP develops, the voltage-dependence of HCN channels shifts mode to one in which activation occurs at more negative potentials. Thus, HCN currents are not readily activated during repolarization. Instead, the activation of the channels is delayed. In the meantime, as the membrane becomes negative, the voltage-dependence for HCN-current activation switches to more positive voltages. At this point, HCN reactivates and currents drive depolarization, so the cycle starts anew.

In the previous two cases, the observance of DVD in the activity of K_V_3.1b and HCN channels guarantees that they are activated when they are “most needed.” A slightly different strategy seems to have evolved with members of the K_V_7 family. For both the homomeric K_V_7.2 and the heteromeric K_V_7.2/K_V_7.3 channels, the deactivation rate decreases as the channels are held activated. However, the response of these channels to depolarization is slower than the duration of the stereotypical neuronal AP (Corbin-Leftwich et al., 2016). Furthermore, the time required to observe a change in the deactivation rate is in the order of tens of milliseconds. Thus, it seems that hysteresis would not have a major role in the response of these channels to the AP under physiological conditions. Instead, the role of hysteresis seems to be at steady state. K_V_7.2 and K_V_7.2/K_V_7.3 channels display meaningful activity at the typical resting membrane potentials (between −40 and −60 mV) (Jentsch, 2000; Cooper, 2012). In steady state at these potentials, the homomeric K_V_7.2 and the heteromeric K_V_7.2/K_V_7.3 channels change their mode of activation so that their deactivation becomes much slower (∼7-fold) than that of channels activated by depolarizing pulses, even those as strong as +40 mV in amplitude with a duration of 100 ms (Corbin-Leftwich et al., 2016). Thus, hysteresis plays a role in stabilizing open channels so that they will be resilient to deactivation when they are the most needed.

## Stochasticity, Reversibility, and Hysteresis

Since the future behavior of a hysteretic system is affected by the past, this implies that voltage-gated channels displaying hysteresis will “remember” what happened in the past. As a result, they are said to have a short-term “memory.” The idea that hysteresis confers “memory” to a system directly collides with two well-accepted concepts in ion channel function: 1) that channels seems to behave stochastically, and 2) that channel activity is thermodynamically reversible (except for the fibrosis transmembrane conductance regulator and similar molecules) (Csanady et al., 2010). One of the fundamental assumptions regarding the first concept is that channels adopt discrete states—transitions between these states depend only on the current state (Colquhoun and Hawkes, 1977). In other words, transitions are “memoryless” events. This property seems to sit diametrically opposite from the process of hysteresis that have been discussed above.

However, these two types of process are compatible. The “lack of memory” of a stochastic process suggests that the current state is the only influence on the probability of a channel to transition states and on what that new state would be—what happened in the past is irrelevant (Colquhoun and Hawkes, 1977). However, the presence of hysteresis does not rely on whether the transition possesses memory or not. Instead, hysteresis exists when there is more than one pathway from one set of states to another, where one set of states can be made of a single state (Villalba-Galea, 2017).

**FIGURE 2:**
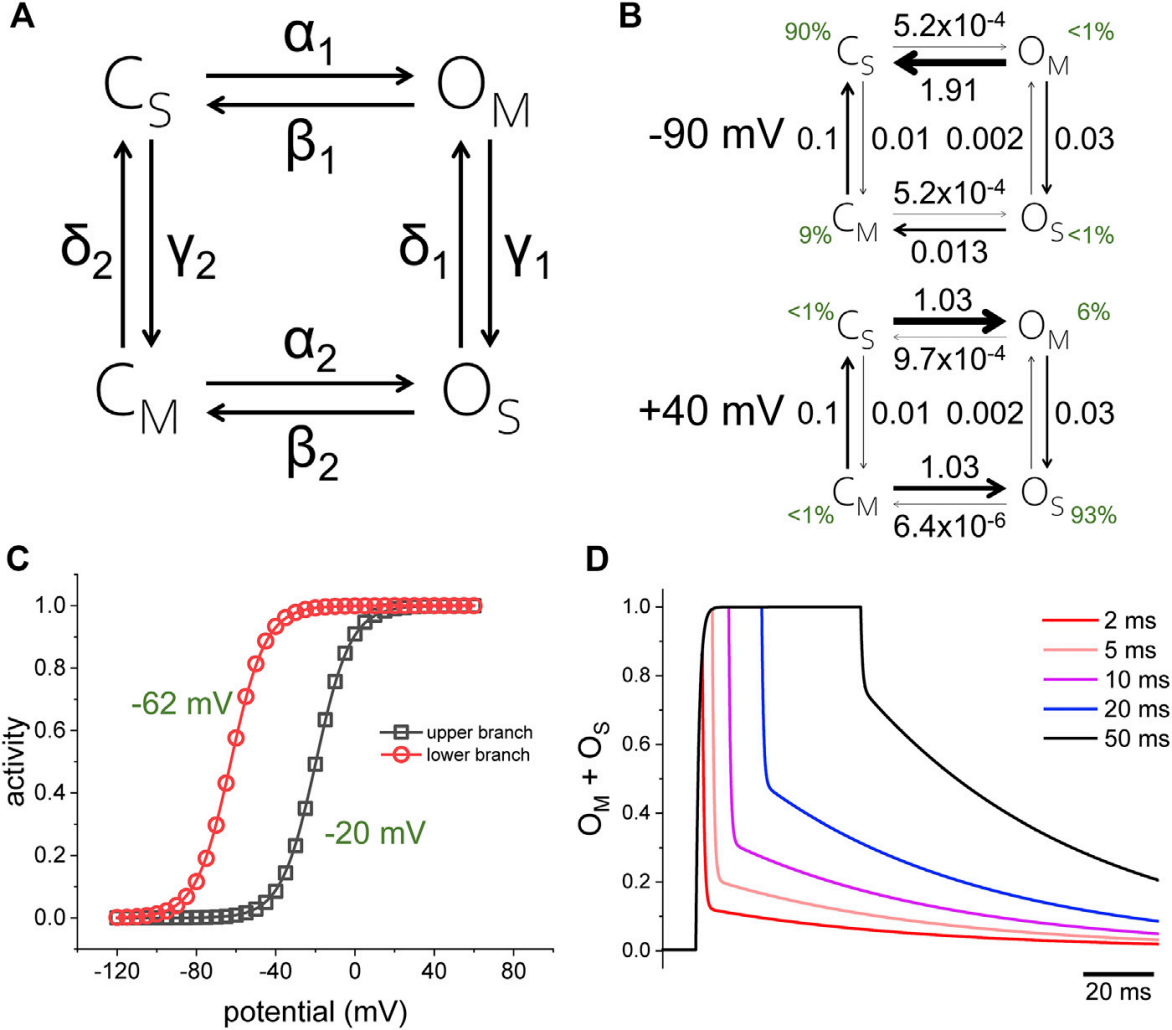
**(A)** Four-state model for channel activity displaying hysteresis. The rates *α* and *β* are functions of the membrane potential; the rates *γ* and *δ* are voltage independent. The top and bottom branches of the model as partially isolated from each other by making the rates *γ* and *δ* small and constant. **(B)** Voltage-dependence for the top and bottom branch when calculated in isolation (making rates *γ* and *δ* equal to zero). **(C)** Rate constants for the model when the membrane potential was either −90 mV **(top)** or +40 mV **(bottom)**. Rate coefficients can be found in **Table 1**. **(D)** Simulation of the model applying +40-mV pulses of different duration (2, 5, 10, 20, and 50 ms). As the activation was prolonged, the temporal profile of deactivation changed, becoming slower. The rates *α*_i_ and *β*_i_ were defined by the functions $\alpha_{i}=\alpha_{0,i}e^{1.5FV/RT}$ and $\beta_{i}=\beta_{0,i}e^{-1.5FV/RT}$, respectively. The model assumes a charge of 1.5 e- associated with each voltage-dependent rate.

To illustrate how a stochastic process can also display hysteresis, let us consider a model containing two sets of closed and open states, C and O, which are alternatingly stable (subscripted with a “S”) and meta-stable (subscripted with a “M”) (Figure 2A, rate coefficients in Table 1). The potential of half-maximum activity (V_h_) for the top branch of the model is −20 mV; for the bottom branch, V_h_ is −65 mV (Figures 2A,B). Both branches are connected through voltage independent transitions. In steady state at −90 mV, the rates favor the population of states C_S_ and C_M_, with C_S_ constituting 90% of the population (Figure 2C, top, green digits). On the other hand, in steady state at +40 mV, state O_M_ and O_S_ are most favored, with O_S_ making up 93% of the population (Figure 2C, bottom, green digits). To reach state O_S_ during activation, the model must go through either C_M_ or O_M_. At +40 mV, the rate *α*_1_ is 2 orders of magnitude larger than the rate γ_1_, thus the activation preferentially proceeds through the top branch. When returning to −90 mV, the rate *β*_2_ is six times larger than δ1, thus deactivation proceeds preferentially through the lower branch in a 1 to 7 ratio. This indicates that this stochastic model follows a preferred direction. This explanation applies only to channels that are in steady state. The situation is different in channels before reaching steady state. To illustrate this idea, let us consider that deactivation in the top branch is 146 times faster than in the bottom branch. Following activation at +40 mV, the overall speed of deactivation changes as a function of the time that the model was kept activated. This model shows that prolonging the duration of the activation results in a decrease in the deactivation kinetics, as deactivation from O_M_ is faster than from O_S_.

**TABLE 1:** Rate coefficients for the four-state model in **Figure 2**.

Coefficient	Value (ms^−1^)	Coefficient	Value (ms^−1^)
*α*_0,1_	0.100	*α*_0,2_	0.1000
*β*_0,1_	0.010	*β*_0,2_	0.0001
*γ*_1_	0.030	*γ*_2_	0.0100
*δ*_1_	0.002	*δ*_2_	0.1000

Another important issue is reversibility. In the case of the hypothetical model considered here (Figure 2), reversibility is guaranteed, as the product of the coefficient rates in one direction of the cycle is equal to the coefficient rate in the opposite direction (Onsager, 1931). In addition, the total sensing charge mobilized in the top branch is equal to the total sensing charge mobilized in the bottom branch. Therefore, the example presented here is a stochastic reversible model that presents a preferred directing and changing kinetic of deactivation as a function of the duration of activation.

## Hysteresis and Channel Structures

Structural models of voltage-gated channels obtained from crystallography studies likely represent stable conformations of these proteins in the absence of an electric field (at 0 mV). Furthermore, the VSD in such models may likely be in a relaxed state of the domain. This implies that structures which are commonly regarded to be representing voltage-gated channels in activated states may likely instead be representing channels in a conformation that is not the “short-lived” (meta-stable) open state. This would have implications in our understanding of the intramolecular interactions governing the activity of channels and of the intermolecular interactions of channels with modulatory subunits, signaling molecules, drugs, and other factors affecting their activity.

## A Final Thought

In general, hysteresis seems to dynamically adjust the voltage-sensitivity and kinetics to optimize channel function to match its physiological role. DVD, an example of hysteresis, may be mechanistically rooted in VSD relaxation and other processes found in the pore domain, intracellular domains, and auxiliary subunits. Although discussing these instances is beyond the scope of this review, it is important to mention a few examples for a more complete understanding. These examples include: hysteresis in the proton-dependent activation of KcsA (Tilegenova et al., 2017), hysteresis in the binding of nucleotides in CNG channels (Nache et al., 2013), and hysteresis in the activity of TRP channels (Liu et al., 2011).

## Author Contributions

CV-G wrote the review article. AC contributed additional research content and improved interpretation of the writing.

## Conflict of Interest

The authors declare that the research was conducted in the absence of any commercial or financial relationships that could be construed as a potential conflict of interest.

## References

[B1] Ahern C. A. Horn R. (2005). Focused electric field across the voltage sensor of potassium channels. Neuron 48 (1), 25–29. 10.1016/j.neuron.2005.08.020 16202706

[B2] Akemann W. Lundby A. Mutoh H. Knöpfel T. (2009). Effect of voltage sensitive fluorescent proteins on neuronal excitability. Biophys. J. 96 (10), 3959–3976. 10.1016/j.bpj.2009.02.046 19450468 PMC2712148

[B3] Bezanilla F. Taylor R. E. Fernández J. M. (1982). Distribution and kinetics of membrane dielectric polarization. 1. Long-term inactivation of gating currents. J. Gen. Physiol. 79 (1), 21–40. 10.1085/jgp.79.1.21 7061986 PMC2215491

[B4] Bruening-Wright A. Larsson H. P. (2007). Slow conformational changes of the voltage sensor during the mode shift in hyperpolarization-activated cyclic-nucleotide-gated channels. J. Neurosci. 27 (2), 270–278. 10.1523/JNEUROSCI.3801-06.2007 17215386 PMC6672073

[B5] Chowdhury S. Chanda B. (2012). Estimating the voltage-dependent free energy change of ion channels using the median voltage for activation. J. Gen. Physiol. 139 (1), 3–17. 10.1085/jgp.201110722 22155736 PMC3250103

[B6] Colquhoun D. Hawkes A. G. (1977). Relaxation and fluctuations of membrane currents that flow through drug-operated channels. Proc. R. Soc. Lond. B Biol. Sci. 199 (1135), 231–262. 10.1098/rspb.1977.0137 22856

[B7] Cooper E. C. (2012). “Potassium channels (including KCNQ) and epilepsy,” in Jasper’s basic mechanisms of the epilepsies. 4th Edn Editors Noebels J. L. Avoli M. Rogawski M. A. Olsen R. W. Delgado-Escueta A. V. (Bethesda, MD: National Center for Biotechnology Information (US)). Available from: https://www.ncbi.nlm.nih.gov/books/NBK98164/ 22787644

[B8] Corbin-Leftwich A. Mossadeq S. M. Ha J. Ruchala I. Le A. H. N. Villalba-Galea C. A. (2016). Retigabine holds KV7 channels open and stabilizes the resting potential. J. Gen. Physiol. 147 (3), 229–241. 10.1085/jgp.201511517 26880756 PMC4772374

[B9] Csanady L. Vergani P. Gadsby D. C. (2010). Strict coupling between CFTR’s catalytic cycle and gating of its Cl^−^ ion pore revealed by distributions of open channel burst durations. Proc. Natl. Acad. Sci. U. S. A. 107 (3), 1241–1246. 10.1073/pnas.0911061107 19966305 PMC2824283

[B10] Elinder F. Männikkö R. Pandey S. Larsson H. P. (2006). Mode shifts in the voltage gating of the mouse and human HCN2 and HCN4 channels. J Physiol. 575 (Pt 2), 417–431. 10.1113/jphysiol.2006.110437 16777944 PMC1819464

[B11] Ewing J. A. (1882). On the production of transient electric currents in iron and steel conductors by twisting them when magnetised or by magnetising them when twisted. Proc. R. Soc. Lond. 33, 21–23. 10.1098/rspl.1881.0067

[B12] Hobiger K. Utesch T. Mroginski M. A. Friedrich T. (2012). Coupling of Ci-VSP modules requires a combination of structure and electrostatics within the linker. Biophys. J. 102 (6), 1313–1322. 10.1016/j.bpj.2012.02.027 22455914 PMC3309284

[B13] Hobiger K. Utesch T. Mroginski M. A. Seebohm G. Friedrich T. (2013). The linker pivot in Ci-VSP: the key to unlock catalysis. PLoS One. 8 (7), e70272 10.1371/journal.pone.0070272 23922964 PMC3726396

[B50] Hodgkin A. L. Huxley A. F. (1952). A quantitative description of membrane current and its application to conduction and excitation in nerve. J. Physiol. 117 (4), 500–544. 10.1113/jphysiol.1952.sp004764 12991237 PMC1392413

[B14] Islas L. D. (2016). Functional diversity of potassium channel voltage-sensing domains. Channels 10 (3), 202–213. 10.1080/19336950.2016.1141842 26794852 PMC4954576

[B15] Jentsch T. J. (2000). Neuronal KCNQ potassium channels: physislogy and role in disease. Nat. Rev. Neurosci. 1 (1), 21–30. 10.1038/35036198 11252765

[B16] Kaczmarek L. K. Zhang Y. (2017). Kv3 channels: enablers of rapid firing, neurotransmitter release, and neuronal endurance. Physiol. Rev. 97 (4), 1431–1468. 10.1152/physrev.00002.2017 28904001 PMC6151494

[B17] Kohout S. C. Bell S. C. Liu L. Xu Q. Minor D. L. Jr. Isacoff E. Y. (2010). Electrochemical coupling in the voltage-dependent phosphatase Ci-VSP. Nat. Chem. Biol. 6 (5), 369–375. 10.1038/nchembio.349 20364128 PMC2857593

[B18] Labro A. J. Lacroix J. J. Villalba-Galea C. A. Snyders D. J. Bezanilla F. (2012). Molecular mechanism for depolarization-induced modulation of Kv channel closure. J. Gen. Physiol. 140 (5), 481–493. 10.1085/jgp.201210817 23071266 PMC3483114

[B19] Labro A. J. Priest M. F. Lacroix J. J. Snyders D. J. Bezanilla F. (2015). Kv3.1 uses a timely resurgent K^+^ current to secure action potential repolarization. Nat. Commun. 6, 10173 10.1038/ncomms10173 26673941 PMC4703866

[B20] Lacroix J. J. Labro A. J. Bezanilla F. (2011). Properties of deactivation gating currents in Shaker channels. Biophys. J. 100 (5), L28–L30. 10.1016/j.bpj.2011.01.043 21354387 PMC3043211

[B21] Lien C.-C. Jonas P. (2003). Kv3 potassium conductance is necessary and kinetically optimized for high-frequency action potential generation in hippocampal interneurons. J. Neurosci. 23 (6), 2058–2068. 10.1523/jneurosci.23-06-02058.2003 12657664 PMC6742035

[B22] Liu B. Yao J. Zhu M. X. Qin F. (2011). Hysteresis of gating underlines sensitization of TRPV3 channels. J. Gen. Physiol. 138 (5), 509–520. 10.1085/jgp.201110689 22006988 PMC3206302

[B23] Männikkö R. Pandey S. Larsson H. P. Elinder F. (2005). Hysteresis in the voltage dependence of HCN channels: conversion between two modes affects pacemaker properties. J. Gen. Physiol. 125 (3), 305–326. 10.1085/jgp.200409130 15710913 PMC2234019

[B24] Murata Y. Iwasaki H. Sasaki M. Inaba K. Okamura Y. (2005). Phosphoinositide phosphatase activity coupled to an intrinsic voltage sensor. Nature 435 (7046), 1239–1243. 10.1038/nature03650 15902207

[B25] Murata Y. Okamura Y. (2007). Depolarization activates the phosphoinositide phosphatase Ci-VSP, as detected in *Xenopus* oocytes coexpressing sensors of PIP_2_ . J. Physiol. 583 (Pt 3), 875–889. 10.1113/jphysiol.2007.134775 17615106 PMC2277204

[B26] Nache V. Eick T. Schulz E. Schmauder R. Benndorf K. (2013). Hysteresis of ligand binding in CNGA_2_ ion channels. Nat. Commun. 4, 2866 10.1038/ncomms3866 24287615 PMC3868267

[B27] Olcese R. Latorre R. Toro L. Bezanilla F. Stefani E. (1997). Correlation between charge movement and ionic current during slow inactivation in Shaker K^+^ channels. J. Gen. Physiol. 110 (5), 579–589. 10.1085/jgp.110.5.579 9348329 PMC2229383

[B28] Onsager L. (1931). Reciprocal relations in irreversible processes. I. Phys. Rev. 37 (4), 405–426. 10.1103/PhysRev.37.405

[B29] Pennefather P. S. Zhou W. DeCoursey T. E. (1998). Idiosyncratic gating of HERG-like K^+^ channels in microglia. J. Gen. Physiol. 111 (6), 795–805. 10.1085/jgp.111.6.795 9607937 PMC2217153

[B30] Piper D. R. Varghese A. Sanguinetti M. C. Tristani-Firouzi M. (2003). Gating currents associated with intramembrane charge displacement in HERG potassium channels. Proc. Natl. Acad. Sci. U. S. A. 100 (18), 10534–10539. 10.1073/pnas.1832721100 12928493 PMC193596

[B31] Priest M. F. Lacroix J. J. Villalba-Galea C. A. Bezanilla F. (2013). S3-S4 linker length modulates the relaxed state of a voltage-gated potassium channel. Biophys. J. 105 (10), 2312–2322. 10.1016/j.bpj.2013.09.053 24268143 PMC3838747

[B32] Rudy B. Chow A. Lau D. Amarillo Y. Ozaita A. Saganich M. (1999). Contributions of Kv3 channels to neuronal excitability. Ann. N. Y. Acad. Sci. 868, 304–343. 10.1111/j.1749-6632.1999.tb11295.x 10414303

[B33] Rudy B. McBain C. J. (2001). Kv3 channels: voltage-gated K^+^ channels designed for high-frequency repetitive firing. Trends Neurosci. 24 (9), 517–526. 10.1016/s0166-2236(00)01892-0 11506885

[B34] Sakata S. Okamura Y. (2019). Dynamic structural rearrangements and functional regulation of voltage‐sensing phosphatase. J. Physiol. 597 (1), 29–40. 10.1113/JP274113 30311949 PMC6312414

[B35] Shi Y. P. Thouta S. Cheng Y. M. Claydon T. W. (2019). Extracellular protons accelerate hERG channel deactivation by destabilizing voltage sensor relaxation. J. Gen. Physiol. 151 (2), 231–246. 10.1085/jgp.201812137 30530765 PMC6363419

[B36] Shi Y. P. Thouta S. Claydon T. W. (2020). Modulation of hERG K^+^ channel deactivation by voltage sensor relaxation. Front. Pharmacol. 11, 139 10.3389/fphar.2020.00139 32184724 PMC7059196

[B37] Starace D. M. Bezanilla F. (2001). Histidine scanning mutagenesis of basic residues of the S4 segment of the Shaker K^+^ channel. J. Gen. Physiol. 117 (5), 469–490. 10.1085/jgp.117.5.469 11331357 PMC2233663

[B38] Starace D. M. Bezanilla F. (2004). A proton pore in a potassium channel voltage sensor reveals a focused electric field. Nature 427 (6974), 548–553. 10.1038/nature02270 14765197

[B39] Starace D. M. Stefani E. Bezanilla F. (1997). Voltage-dependent proton transport by the voltage sensor of the Shaker K^+^ channel. Neuron 19 (6), 1319–1327. 10.1016/s0896-6273(00)80422-5 9427254

[B40] Thouta S. Hull C. M. Shi Y. P. Sergeev V. Young J. Cheng Y. M. (2017). Stabilization of the activated hERG channel voltage sensor by depolarization involves the S4-S5 linker. Biophys. J. 112 (2), 300–312. 10.1016/j.bpj.2016.12.021 28122216 PMC5266146

[B41] Tilegenova C. Cortes D. M. Cuello L. G. (2017). Hysteresis of KcsA potassium channel’s activation- deactivation gating is caused by structural changes at the channel’s selectivity filter. Proc. Natl. Acad. Sci. U. S. A. 114 (12), 3234–3239. 10.1073/pnas.1618101114 28265056 PMC5373385

[B42] Villalba-Galea C. A. (2012a). New insights in the activity of voltage sensitive phosphatases. Cell. Signal. 24 (8), 1541–1547. 10.1016/j.cellsig.2012.03.013 22481094

[B43] Villalba-Galea C. A. (2012b). Voltage-controlled enzymes: the new *Janus Bifrons* . Front. Pharmacol. 3, 161 10.3389/fphar.2012.00161 22993507 PMC3440755

[B44] Villalba-Galea C. A. (2017). Hysteresis in voltage-gated channels. Channels 11 (2), 140–155. 10.1080/19336950.2016.1243190 27689426 PMC5398603

[B45] Villalba-Galea C. A. Miceli F. Taglialatela M. Bezanilla F. (2009a). Coupling between the voltage-sensing and phosphatase domains of Ci-VSP. J. Gen. Physiol. 134 (1), 5–14. 10.1085/jgp.200910215. 19564425 PMC2712979

[B46] Villalba-Galea C. A. Sandtner W. Dimitrov D. Mutoh H. Knöpfel T. Bezanilla F. (2009b). Charge movement of a voltage-sensitive fluorescent protein. Biophys. J. 96 (2), L19–L21. 10.1016/j.bpj.2008.11.003 19167283 PMC2716470

[B47] Villalba-Galea C. A. Sandtner W. Starace D. M. Bezanilla F. (2008). S4-based voltage sensors have three major conformations. Proc. Natl. Acad. Sci. U. S. A. 105 (46), 17600–17607. 10.1073/pnas.0807387105 18818307 PMC2584729

[B48] Zhao J. Blunck R. (2016). The isolated voltage sensing domain of the Shaker potassium channel forms a voltage-gated cation channel. Elife 5, e18130 10.7554/eLife.18130 27710769 PMC5092046

